# Eliminating Stubborn Insulated Deposition by Coordination Effect to Boost Zn Electrode Reversibility in Aqueous Electrolyte

**DOI:** 10.3389/fchem.2022.851973

**Published:** 2022-03-15

**Authors:** Yuzhuo Jiang, Xinyao Xia, Siyi Qian, Jing Zhang, Pinxin Zhou, Xuefang Gu, Shu Tian, Yijun Qian, Haoqing Ji, Jie Liu, Tao Qian

**Affiliations:** ^1^ School of Chemistry and Chemical Engineering, Nantong University, Nantong, China; ^2^ College of Energy, Key Laboratory of Advanced Carbon Materials and Wearable Energy Technologies of Jiangsu Province, Soochow University, Suzhou, China; ^3^ State Key Laboratory of Space Power-Sources Technology, Shanghai Institute of Space Power-Sources, Shanghai, China; ^4^ Institute for Frontier Materials, Deakin University, Victoria, China

**Keywords:** aqueous zinc-ion batteries, electrolyte additive, coordination effect, interfacial by-products, insulating layer

## Abstract

Aqueous rechargeable zinc-ion batteries (ZIBs) have recently shined in energy storage and transmission, which are due to high safety and low cost. However, the extremely stubborn by-products in the Zn anode severely inhibited the Zn^2+^ adsorption/desorption and exacerbated the dendrite formation. Herein, we report a facile strategy to eliminate inert Zn_4_(OH)_6_SO_4_·xH_2_O for the improvement of ZIBs according to the coordination effect by employing ethylenediaminetetraacetic acid-diamine (EDTA-2Na) as a coordination additive in traditional electrolyte. Zn^2+^ is coordinated with the carboxyl group of the four acetyl carboxyl groups and the N in C–N bonds, forming a new chelating structure, and thus stubborn deposition will be dissolved in the electrolyte. As a result, the discharge capacity of 102 mAh g^−1^ in the ZnSO_4_/Li_2_SO_4_ with EDTA-2Na electrolyte at a current density of 4 C and a stable cycle life with a capacity of 90.3% after 150 cycles are achieved. It has been concluded that the coordination effect strategy provides a valuable idea for solving the defects of ZIBs.

## Introduction

The huge advantages in energy density, cycle stability, and output voltage make lithium-ion batteries (LIBs) available and popular (Clément et al., 2020; Liu et al., 2021; Zhang et al., 2021), while frequent reports on fire and explosion of LIBs, due to the flammability of organic electrolytes, raised people’s concerns on their safety (Zhu et al., 2021; Xu and Jiang, 2021). Aqueous rechargeable zinc-ion batteries (ZIBs) with high theoretical capacities (volumetric capacity of 5,855 mA h cm^−3^ and gravimetric capacity of 820 mA h g^−1^), low cost, and absolute security characteristics are practical alternatives (Liu et al., 2019; Qiu et al., 2019; Wu H Y et al., 2021). However, ZIBs face a series of severe challenges especially for zinc anodes, including dendrite growth and related parasitic reactions caused by free water (such as HER and by-product) (Yang et al., 2020; Sun H et al., 2021). Many methods have been reported to improve the performance of ZIBs by inhibiting hydrogen evolution or dendrite in aqueous electrolytes and proved to be effective, such as electrolyte additives (Soundharrajan et al., 2020; Guo et al., 2021; Hao et al., 2021; Guan et al., 2022), artificial SEI layers (Hao et al., 2020; Di et al., 2021; Hong et al., 2021; Shin et al., 2021), and zinc anode modification (Yang et al., 2021; Zhang et al., 2021; Zhang et al., 2021; Zhou et al., 2021). Nevertheless, the side reactions between Zn and aqueous electrolyte have rarely been paid attention to, which closely caused the decreased capacity and poor stability of the battery.

In the local alkaline environment caused by the hydrogen evolution, Zn electrode would be corroded by increased concentration of hydroxide ions to generate ionic-insulating Zn_4_SO_4_(OH)_6_·xH_2_O, which becomes the barrier for ion/electron diffusion, such as Eqs 1, 2 (Cao Z et al., 2020).

$$2H_{2}O\left(l\right)+2e^{-}\to H_{2}\left(g\right)+2OH^{-}\left(aq\right)$$ (1)

$$4Zn^{2+}\left(aq\right)+6OH^{-}\left(aq\right)+SO_{4}^{2-}\left(aq\right)\to ZnSO_{4}{\left(OH\right)}_{6}\left(s\right)$$ (2)

The inert and stubborn depositions would adhere to the Zn anode, deteriorate electrical contact, and severely attenuate the capacity, which seriously hinders the commercialization of ZIBs (Pang et al., 2020; Sun H et al., 2021). In the water-based electrolyte, the insoluble passivation layer seriously affects the transfer of solvated zinc, which greatly reduces the capacity and life of the battery (Xu and Jiang, 2021a; Du et al., 2021). Unfortunately, once this insulating passivation layer has been deposited on the zinc anode, the Zn plating/stripping performance of ZIBs will drastically decrease due to the increase in charge transfer resistance (Zhang et al., 2020; Zhang et al., 2021; Du et al., 2022). What is more, owing to part of the electrolyte converted into the insoluble passivation layer, the concentration of the electrolyte becomes unstable with the operation of the battery, and even the water solvent in the electrolyte will continue to decrease due to the continuous generation of the layer, which intensifies the deterioration of the battery (Liu et al., 2021; Song and Zhong, 2021).

**FIGURE 1:**
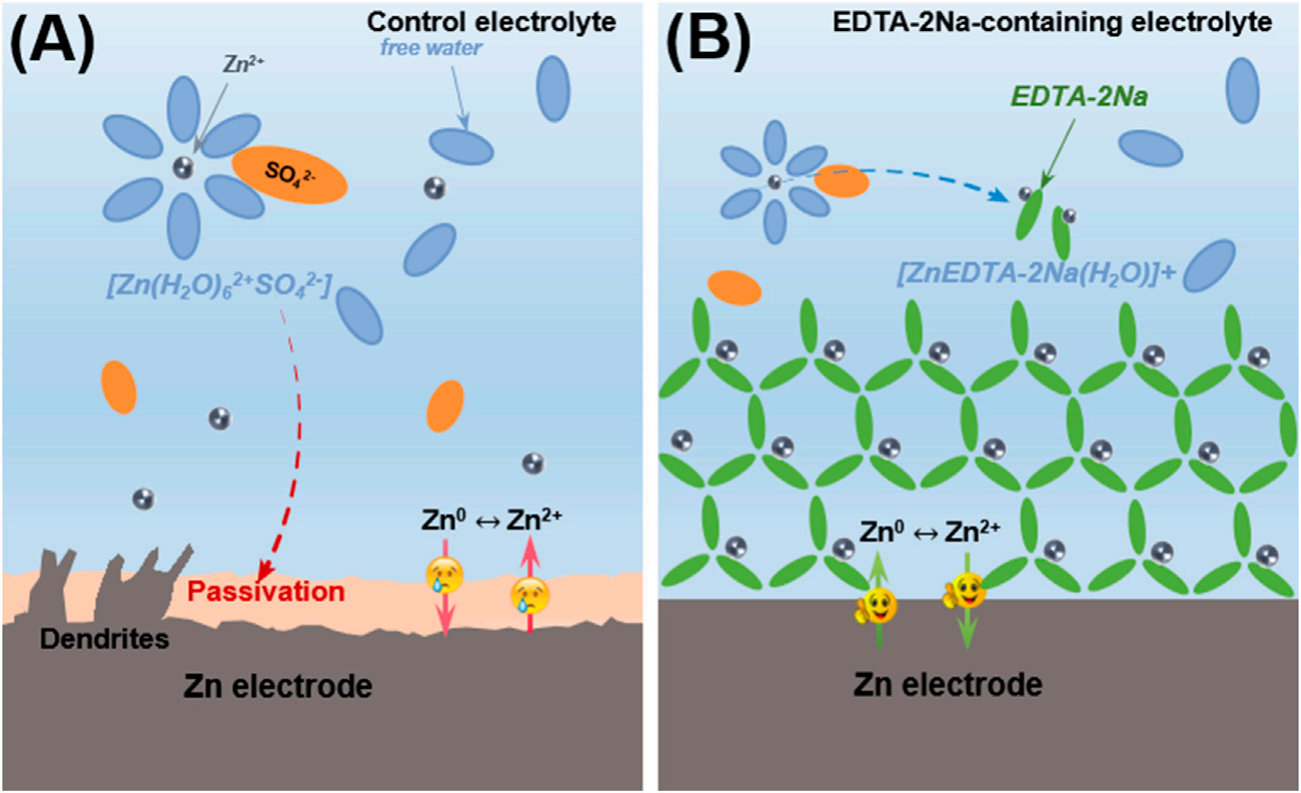
Schematic illustration of Zn surface evolution. **(A)** Stubborn insulated deposition and dendrite formation caused by attack from desolvation process on Zn foil. **(B)** Function mechanism of EDTA-2Na chelating agent to eliminate deposition and forms a stable chelating state in aqueous electrolyte.

Herein, we report a facile strategy to eliminate inert Zn_4_(OH)_6_SO_4_·xH_2_O for the improvement of ZIBs by employing ethylenediaminetetraacetic acid-diamine (EDTA-2Na) as coordination additive in traditional electrolyte (Figures 1A,B). During the charging/discharging process, the carboxyl group of the four acetyl carboxyl groups and the N in C–N bonds will coordinate with Zn^2+^ and a new [ZnEDTA-2Na(H_2_O)]^+^ chelating structure forms in the prepared electrolyte (Cao et al., 2019). In the local alkaline environment generated by the side reaction, the generated basic zinc sulfate will be dissolved in the electrolyte and complexed in the aqueous electrolyte in the form of EDTA-Zn. During the whole process of dezincification and intercalation, Zn^2+^ can be uniformly transferred and deposited in the electrolyte due to the easier de-solvation process, which can be evidenced by lower overpotential during Zn deposition in symmetric battery. The prepared electrolyte significantly improved Zn plating/stripping Coulombic efficiency (CE) to 99.2% at 5 mA cm^−2^ and 2.5 mA h cm^−2^. After 150 cycles, at a current density of 4 C (1 C = 148 mA g^−1^), the discharge capacity is 102 mA h g^−1^, and the capacity retention rate is 90.3%. This optimization strategy for the passivation layer has greatly expanded our thinking and provided inspiration for solving the problem of ZIBs stability.

## Results and Discussion

**FIGURE 2:**
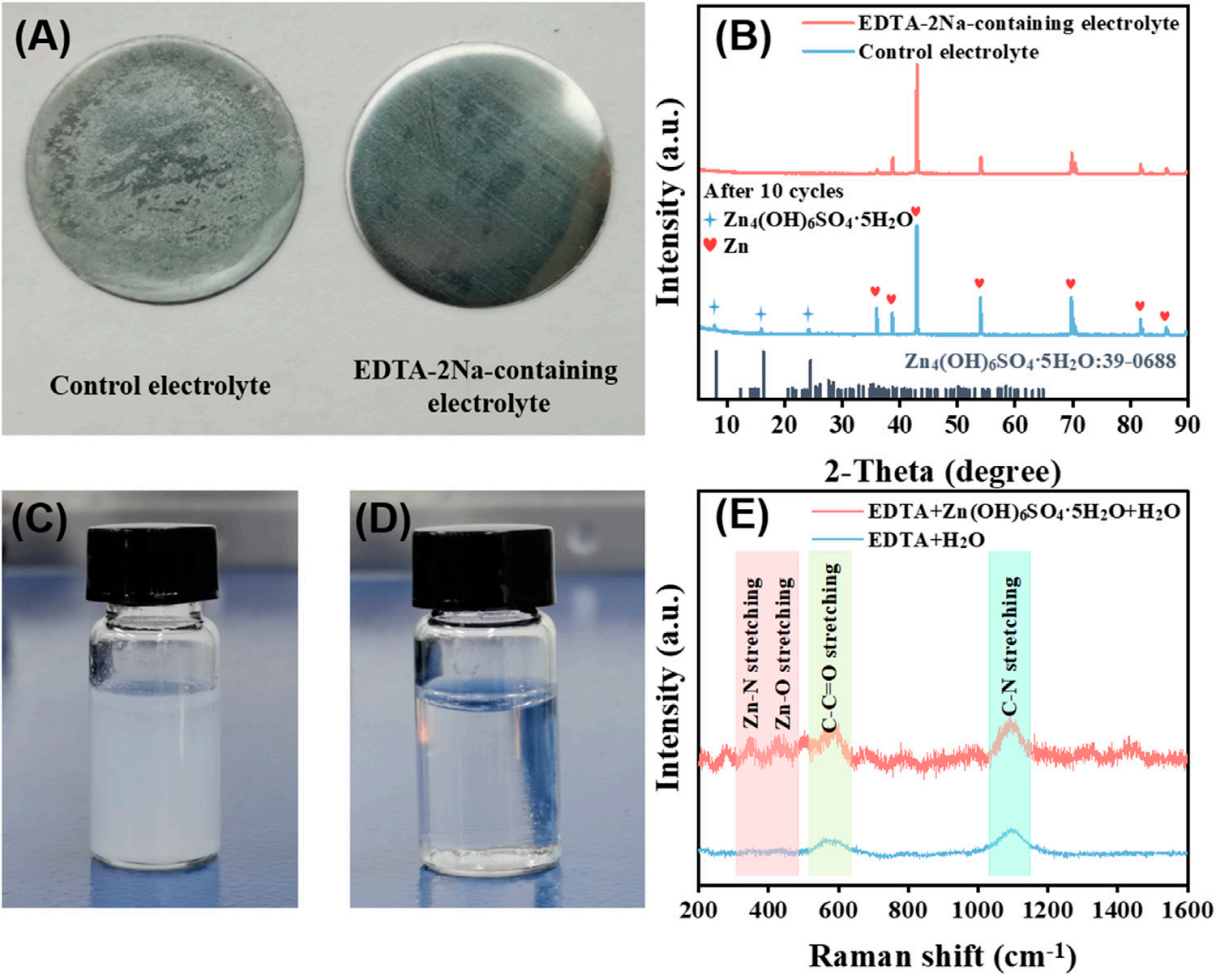
**(A)** Image of Zn foil in control electrolyte and in EDTA-2Na-containing electrolyte. **(B)** XRD of Zn foil in control electrolyte and in EDTA-2Na-containing electrolyte. **(C)** Image of the Zn_4_(OH)_6_SO_4_.5H_2_O suspended in aqueous solution and **(D)** insoluble matter dissolution after adding EDTA-2Na. **(E)** Raman of solution before and after dissolving insoluble Zn_4_(OH)_6_SO_4_.5H_2_O with EDTA-2Na.

The aqueous electrolyte with 1 M ZnSO_4_ and 3 M Li_2_SO_4_ in water is employed as control. As shown in Figure 2A, after cycling in the control electrolyte, the surface of the zinc anode is covered by an insulating layer. The peak at about 9.8^o^ in the x-ray diffraction (XRD) (Figure 2B) spectrum confirms that the insulating layer is Zn_4_(OH)_6_SO_4_.5H_2_O (Jiao et al., 2021). This is due to the hydrogen evolution reaction of water in the aqueous electrolyte causing the partial formation of an alkaline environment in the electrolyte, which reacts with Zn^2+^ to form an insoluble insulating solid precipitation and prevent the transfer of ions and electrons at the interface. As a contrast, the zinc anode cycled in the EDTA-2Na-containing electrolyte exhibits much cleaner surface and the XRD pattern shows no obvious peak at 9.8^o^. The insulating zinc salt Zn_4_(OH)_6_SO_4_.5H_2_O presents fine white granular insoluble matter in the water phase as shown in Figure 2C. After introducing EDTA-2Na, the strong interaction between EDTA anions and Zn^2+^ promotes the dissolution of Zn_4_(OH)_6_SO_4_.5H_2_O. As evidenced in Figure 2D, stubborn Zn_4_(OH)_6_SO_4_.5H_2_O can be dissolved in EDTA-2Na-containing electrolyte. As shown in Figure 2E, the EDTA-2Na solution shows relatively gentle Raman bands in the 300–600 cm^−1^ region, while after introducing Zn_4_(OH)_6_SO_4_.5H_2_O, the mixed solution has fairly obvious peaks in this region (Wu et al., 2015; Liu et al., 2020). The newly appeared Zn–N and Zn–O bonds stretching and bending and other framework vibrations, which is due to the chelation coordination, can well explain the reason for most of the new peaks in the 300–600 cm^−1^. In the presence of Zn^2+^, the typical UV-Vis spectrum of EDTA-Zn has changed markedly, as depicted in Supplementary Figure S1.

**FIGURE 3:**
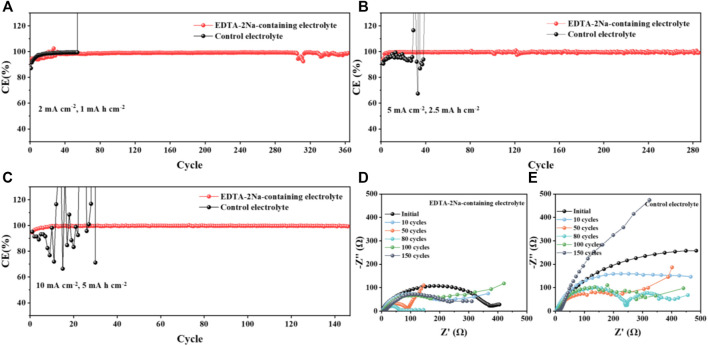
Scheme caption CE of Zn||Cu cells with and without additives of the EDTA-2Na in the control electrolyte cycled under **(A)** 2 mA cm^−2^ and 1 mA h cm^−2^, **(B)** 5 mA cm^−2^ and 2.5 mA h cm^−2^, and **(C)** 10 mA cm^−2^ and 5 mA h cm^−2^ conditions. **(D,E)** EIS plots of Zn||Cu cells with and without EDTA-2Na electrolyte additive after various numbers of cycles.

The extremely uncontrollable insulating layer of water-based batteries seriously affects the Coulombic efficiency of the battery. In order to verify that suppressing the passivation layer can effectively improve the battery efficiency, we first performed Zn plating/stripping Coulomb efficiency tests in different electrolytes at different current densities of Zn||Cu batteries. The effect of electrolyte with different additives has been tested (Supplementary Figure S2). The result shows that 5% cation additive has the best effect. As shown in Figure 3A, the Zn||Cu battery using EDTA-2Na-containing electrolyte is cycled for 360 cycles at 2 mA cm^−2^ and 1 mA h cm^−2^ and shows high reversibility and stability, while the Zn||Cubattery in control electrolyte fails quickly after only 60 cycles. Furthermore, under higher current density of 5 mA cm^−2^, the control battery is damaged after 30 cycles, while the CE of the battery cycled in EDTA-2Na-containing electrolyte is still as high as 99% after 110 cycles (Figure 3B), which indicates the excellent ability of EDTA anions to suppress by-products. The similar conclusion can be obtained when the current density is increased to 10 mA cm^−2^, where high CE can still be maintained over 150 cycles for the Zn||Cu battery in EDTA-2Na-containing electrolyte (Figure 3C). The addition of EDTA-2Na excellently alleviates the problem of low cycle efficiency limited by the by-product passivation layer. Moreover, the Zn||Cu cell using EDTA-2Na-containing electrolyte also has a much lower resistance than that in control electrolyte (Supplementary Figure S3). According to the Electrochemical impedance spectrum (EIS), the Zn||Cu battery cycled in EDTA-2Na-containing electrolyte exhibits gradually decreased impedance and stabilizes after 80 cycles,which is much smaller than the relative impedance of that in control electrolyte, indicating that the growth of the Zn_4_(OH)_6_SO_4_.5H_2_O insulating layer is effectively controlled(Figures 3D,E) (Cang et al., 2019; Zhang et al., 2019). As the stubborn insulated deposition is continuously generated, the resistance will increase sharply. Stable semicircles mean that the resistance tends to be stable, which also proves that the additive has an efficient effect on inhibiting the formation of insulated deposition (Li et al., 2021; Wang et al., 2021).

**FIGURE 4:**
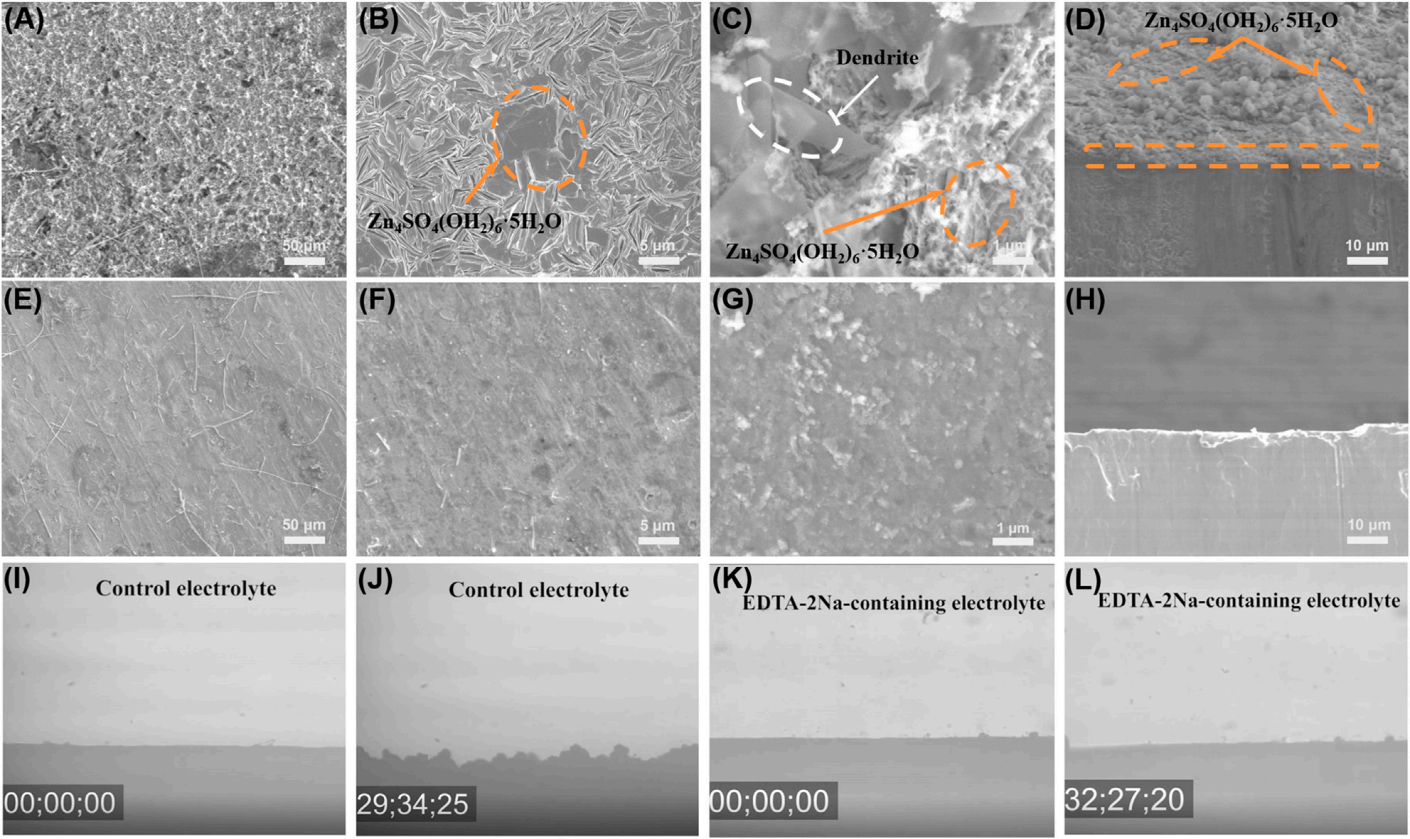
Galvanostatic Zn plating/stripping in Zn||Zn symmetrical cells at 1 mA cm^−2^ and 1 mA h cm^−2^. SEM images of Zn electrodes in Zn||Zn symmetrical cells after 20 plating/striping cycles at 1 mA cm^−2^ and 0.5 mA h cm^−2^ in **(A–D)** control electrolyte and **(E–H)** EDTA-2Na-containing electrolyte. *In situ* optical microscope photos of **(I,J)** control electrolyte and **(K,L)** EDTA-2Na-containing electrolyte.

Zn||Zn symmetric battery was assembled to evaluate the electroplating/stripping stability of Zn under different electrolyte environments. The zinc symmetric battery with EDTA-2Na-containing electrolyte has shown higher reversibility and smaller overpotential (∼30 mV) after 150 h at 1 mA cm^−2^ and 1 mA h cm^−2^ (Supplementary Figure S4). What is more, the depth of discharge (DOD) has been studied by testing Zn||Zn symmetric batteries at 2 mA cm^−2^ for 0.25, 0.5, and 1 h, showing the performance of different DODs. The result has indicated that Zn can stably deposit/strip at 1 mA h cm^−2^, but at higher DODs (2 mA h cm^−2^), voltage fluctuation happens. As a contrast in EDTA-2Na-containing electrolyte, Zn||Zn symmetric battery always exhibits stable Zn deposition/stripping behavior at higher DODs (Hao et al., 2020; Leng et al., 2020; Ma et al., 2020; Hu et al., 2020). The Zn electrode surface morphologies after 20 plating/stripping cycles are characterized using scanning electron microscopy (SEM) (Figure 4). Due to the continuous reaction of Zn with the electrolyte, large-area layered deposits and a large number of flaky dendrites were formed on the Zn surface (Figures 4A–D) in the control electrolyte (Quan et al., 2020; Wu Y et al., 2021). In contrast, the Zn surface in EDTA-2Na-containing electrolyte shows a dense and smooth morphology (Figures 4E–H), confirmed by *in situ* optical microscope test results (Figures 4I–L). Under the *in situ* optical microscope observation, the microscopic phenomenon of zinc surface deposition circulating in the control electrolyte showed uneven, thick and disordered dendrites after about 30 min. On the contrary, the zinc surface observed in the EDTA-2Na-containing electrolyte is smooth and flat without dendrites. The self-healing electrostatic shield effect explains the observed uniform Zn deposition and dendrite suppression (Cao L et al., 2020). It can be obtained from the above content that the introduction of EDTA-2Na significantly improves the Zn plating/stripping capacity and the cycle stability of the zinc anode.

**FIGURE 5:**
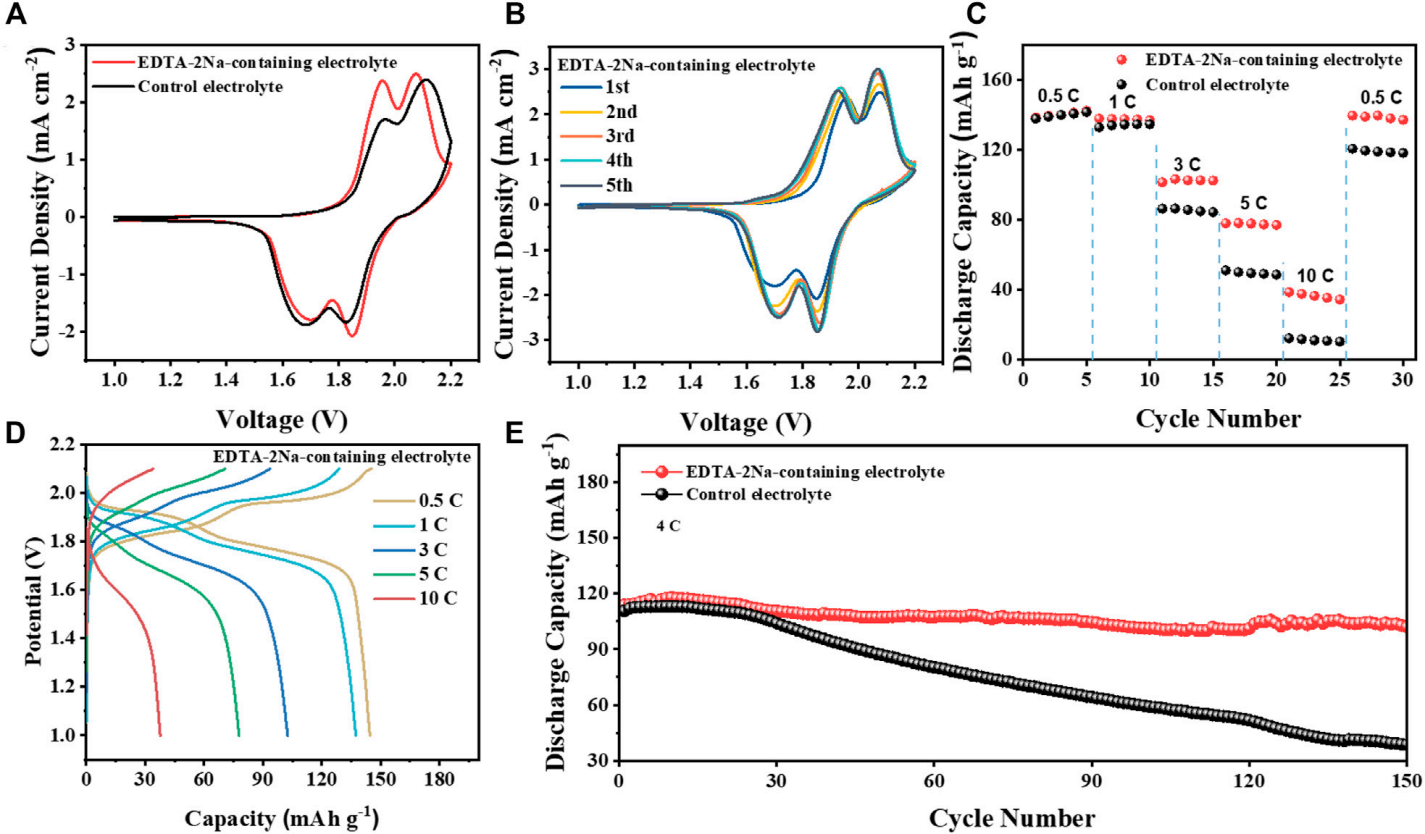
Electrochemical performance of Zn||LiMnO_4_ cells. **(A)** CV of Zn‖LiMnO4 full cells with different electrolytes at a scan rate of 0.5 mV s^−1^. **(B)** CV of Zn||LiMnO_4_ full cells with EDTA-2Na-containing electrolyte for five cycles. **(C)** Rate capability for Zn‖LiMnO_4_ full cells. **(D)** Rate performance in EDTA-2Na-containing electrolyte. **(E)** Cyclic stability and efficiency of Zn||LiMnO_4_ cells in two electrolytes at 4 C.

The EDTA-2Na-containing electrolyte was evaluated in Zn||LiMnO_4_ cell using LiMnO_4_ (LMO) cathodes. Cyclic voltammetry (CV) of Zn||LiMnO_4_ cells in two electrolytes at 0.5 mV s^−1^ is shown in Figure 5A. It was clear that the redox peak gap of the batteries in EDTA-2Na-containing electrolyte was much smaller, indicating that it has a lower overpotential and easier Zn-ion diffusion ability. Compared with the control electrolyte, the CV curves in the EDTA-2Na-containing electrolyte environment have a higher degree of overlap and better stability (Figure 5B and Supplementary Figure S6) The rate performance of LMO cells in EDTA-2Na-containing electrolyte was also evaluated. As in Figures 5C, D and Supplementary Figure S6, the LMO cathodes provided a high capacity of 120 mA h g^−1^ at the rate of 0.5 C, and still maintain 30 mA h g^−1^ even at a high rate of 10 C. The long-term cycling stability of the Zn||LiMnO_4_ cells was evaluated at 4 C in both electrolytes (Figure 5E). The Zn||LiMnO_4_ cells after 150 cycles in EDTA-2Na-containing electrolyte still maintain about 102 mA h g^−1^, which is 90.3% of initial capacity, while the capacity of Zn||LiMnO_4_ cells in control electrolyte rapidly drop to 34.1% of the initial capacity due to the serious by-product passivation layer and zinc dendrites. Meanwhile, some recent relevant and interesting work has been compared, and Supplementary Table S1 has shown specific electrochemical performances. Our work can maintain high capacity retention even at 4 C.

## Conclusion

The stubborn by-product passivation layer and severe zinc dendrite growth are serious challenges for water-based ZIBs. In this work, we use simple addition of EDTA-2Na chelate to control zinc ions in the electrolyte to effectively prevent the formation of the insulating passivation layer and inhibit the growth of zinc dendrites. As a result, this chelate compound dissolves the insoluble matter [Zn_4_(OH)_6_SO_4_.5H_2_O] through strong chelation and complexes with the metal ions in the electrolyte, providing a more orderly and stable plating/stripping environment for Zn^2+^. What is more, the metal cations carried by the chelate salt can effectively inhibit the growth of zinc dendrites, which is attributed to the self-healing electrostatic shield effect exhibited by the carried cations. This subject proves that it is an effective strategy to add chelate salt to aqueous electrolyte, and provides a new idea to eliminate by-products and dendrites to realize industrialized aqueous electrochemical storage equipment.

## Materials and Methods

### Materials

ZnSO_4_.7H_2_O (>99.0%), Li_2_SO_4_·H_2_O (>99.0%), and ethylene diamine tetraacetic acid disodium salt (EDTA-2Na, A.R.> 99.0%) were prepared from Sigma-Aldrich Chemical Co. All reagents were used directly without further purification. All aqueous electrolytes used deionized water as the solvent.

### Characterizations

XRD was carried out on Rigaku Ultima IV. The radiation source was Cu Kα. Samples were scanned at a range of 5°–90° with scan speed 5°min^−1^. SEM images were collected on the GeminiSEM 300 with an accelerating voltage of 5 kV, and it was employed to observe the morphology of anode surface in Zn||Cu batteries after 20 cycles. The Raman spectra were employed by an HR Evolution (HORIBA) confocal Raman spectrometer to obtain the Raman signal of the electrolytes. The *in situ* optical microscope (Caikon DMM-330C) was employed to observe the growth of zinc dendrites in symmetric Zn||Zn batteries.

### Electrochemical Tests

For measurement of Zn CE, a Zn||Cu half-cell was applied. During testing, a given current density and deposition time were used for Zn plating, while a fixed voltage was used to strip the Zn from Cu-foil on the Neware BTS4000 battery test instrument. The Zn||Zn symmetrical battery consists of two zinc sheets and a glass fiber separator with 100 μl of electrolyte that were sandwiched together in a CR2032 coin cell and crimped in the air and were performed in this battery test instrument under different conditions. For the Zn||LiMnO_4_ full cells, LMO electrode and Zn-foil were matched, and glass fiber was used as a separator to assemble the Zn||LMO cells, which were performed on the Neware BTS4000 battery test instrument. CV profiles were performed on an electrochemical station (CHI660E, China) at different scan rates with a voltage range of 1.0–2.2 V, in which EIS was also tested at the voltage of open circuit potential within the frequency range from 10^–2^ to 10^5^ Hz. *Proin nec augue*. The electrolytes used in all types of cell tests are the mixture of 1 M ZnSO_4_.7H_2_O and 3 M Li_2_SO_4_·H_2_O without/with 5% cation additives added. The thickness of Zn anode was ∼0.15 mm. The average mass loading of LMO cathode was ∼2 mg.

## Data Availability

The original contributions presented in the study are included in the article/Supplementary Material. Further inquiries can be directed to the corresponding authors.
