# Supplementary material for: Eliminating Stubborn Insulated Deposition by Coordination Effect to Boost Zn Electrode Reversibility in Aqueous Electrolyte
Source: Front Chem. 2022 Mar 15;10:851973. doi: 10.3389/fchem.2022.851973 (PMC8965326; doi:10.3389/fchem.2022.851973)
Supplement: Supplementary file 3 [file DataSheet1.docx]

***Supplementary Material***

**Eliminating Stubborn Insulated Deposition by Coordination Effect to Boost Zn Electrode Reversibility in Aqueous Electrolyte**

Yuzhuo Jiang^a^, Xinyao Xia^b^, Siyi Qian^a^, Jing Zhang^c^, Pinxin Zhou^c^, Xuefang Gu^a,*^, Shu Tian^a^, Yijin Qian^d,*^, Haoqing Ji^b^, Jie Liu^a,*^, and Tao Qian^a^

^a^ School of Chemistry and Chemical Engineering, Nantong University, Nantong 226019, China

^b^ College of Energy, Key Laboratory of Advanced Carbon Materials and Wearable Energy Technologies of Jiangsu Province, Soochow University, Suzhou 215006, Jiangsu, China

^c^ State Key Laboratory of Space Power-sources Technology, Shanghai Institute of Space Power-Sources, 2965 Dongchuan Road, Shanghai 200245, China.

^d^ Deakin University, Institute for Frontier Materials, Waurn Ponds Campus, Locked Bag 20000, Geelong, Victoria 3220, Australia

E-mail: xuefang818@ntu.edu.cn, qianyij@deakin.edu.au and jliu93@ntu.edu.cn

**Figure S1.** Changes in UV-Vis of EDTA-2Na solution samples with and without Zn_4_(OH)_6_SO_4_·5H_2_O.


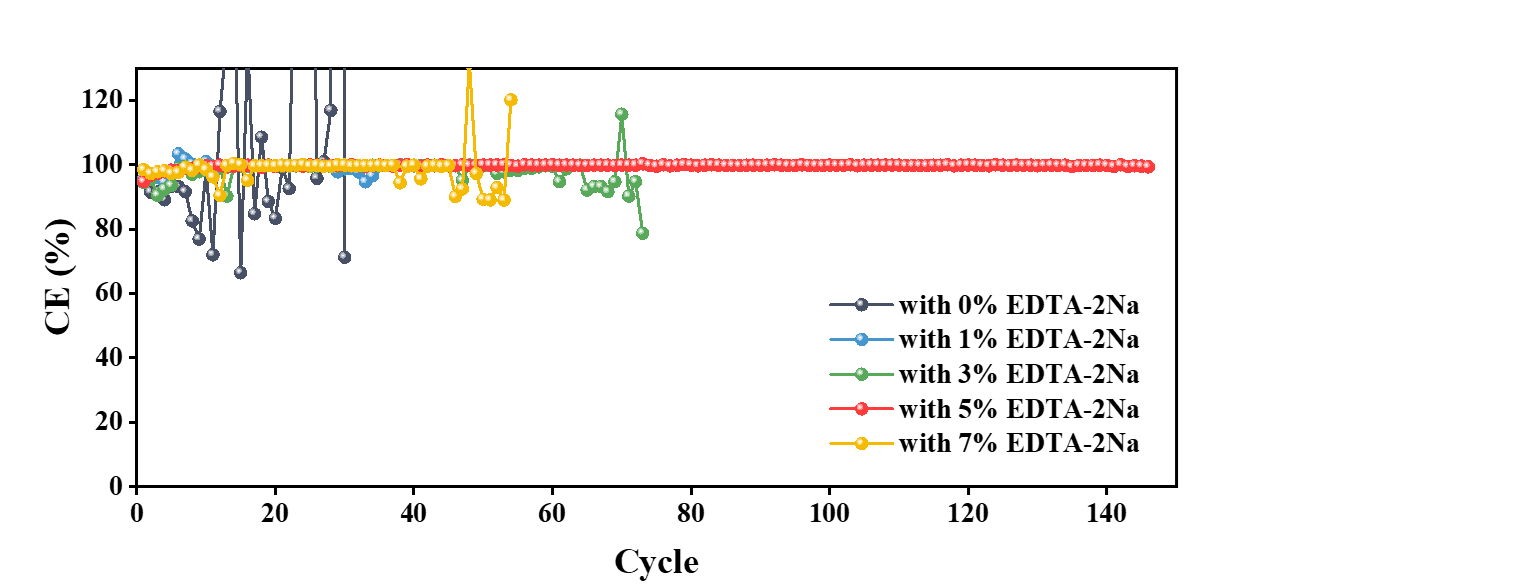


**Figure S2.** Scheme Caption CE of Zn||Cu cells with different mass ratio of EDTA-2Na in the control electrolyte cycled under 10 mA cm^-2^ and 5 mA h cm^-2^ conditions.

**
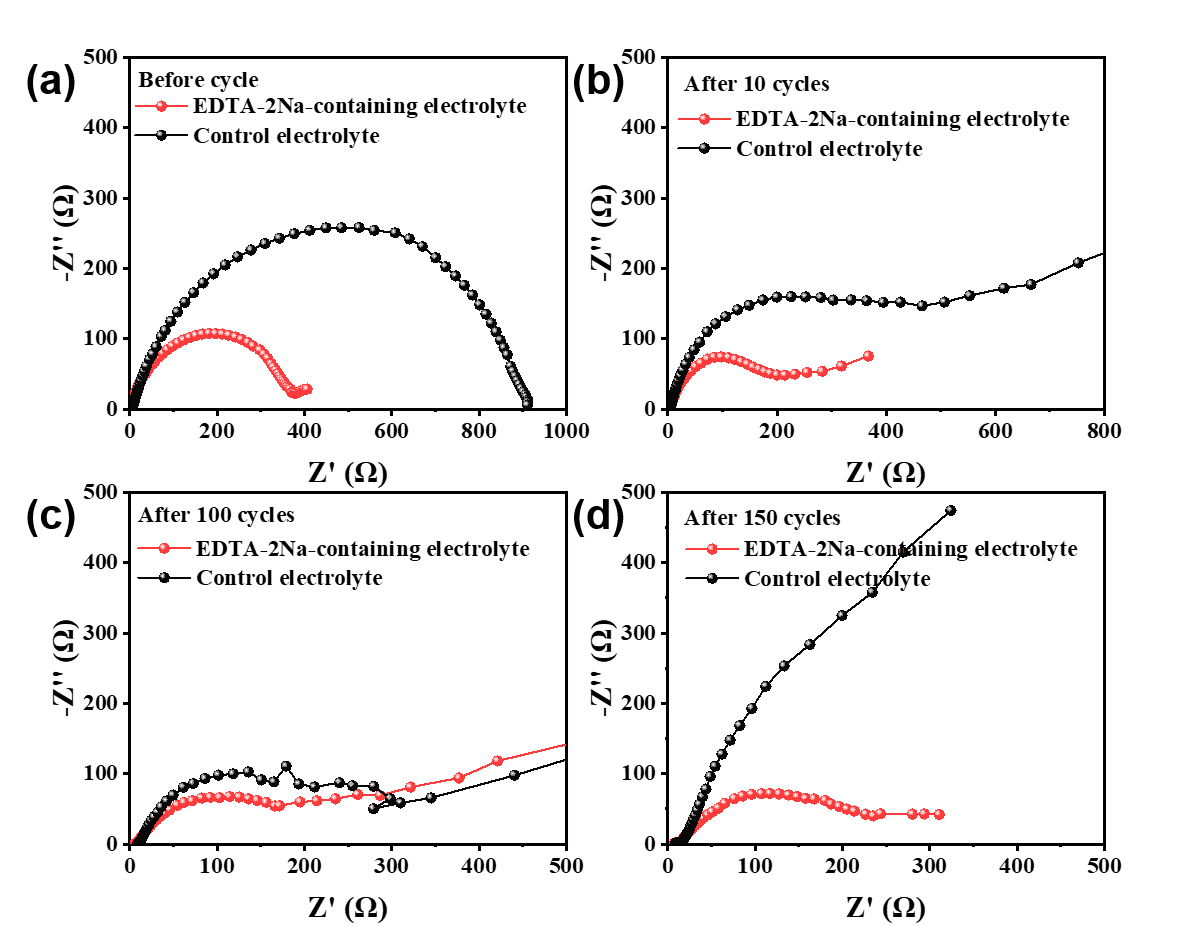
**

**Figure S3.** EIS plots of Zn||Cu cells with and without EDTA-2Na after various numbers of cycles.

**
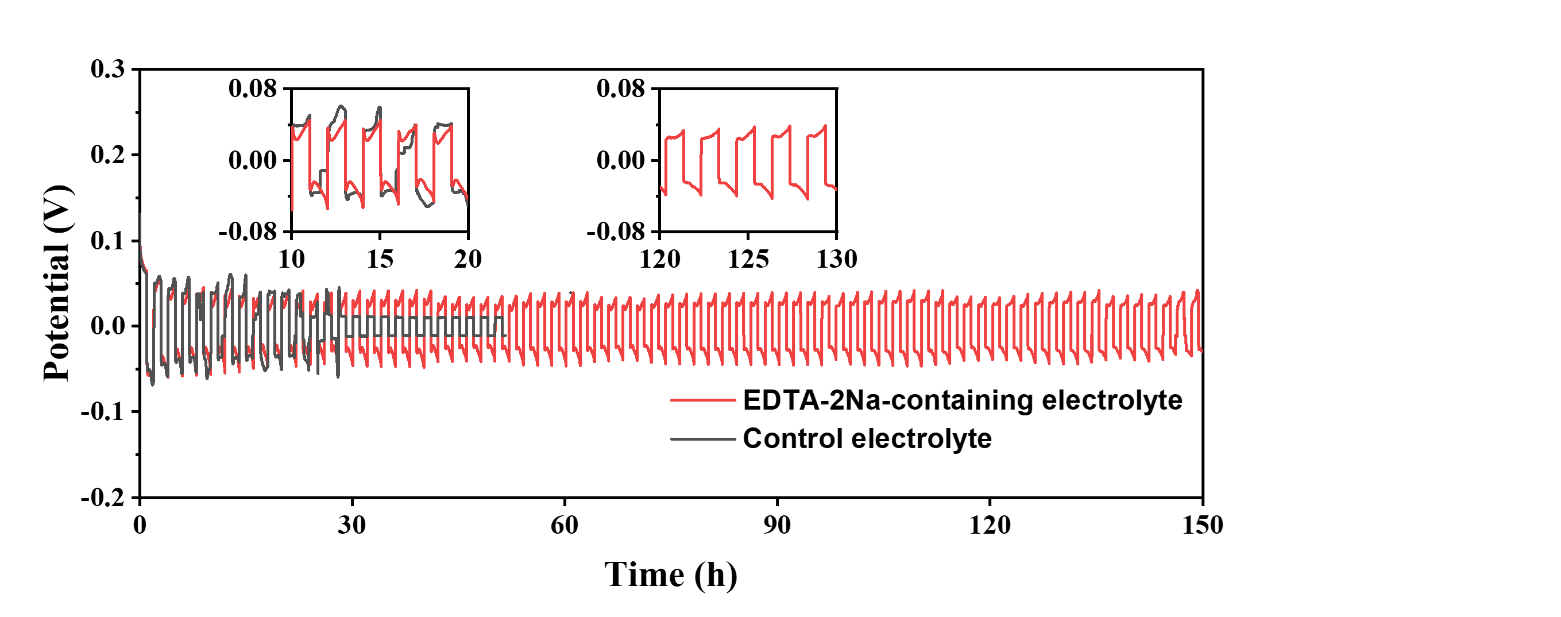
**

**Figure S4.** Galvanostatic Zn plating/stripping in Zn||Zn symmetrical cells at 1 mA cm^-2^ and 1 mA h cm^-2^.

**
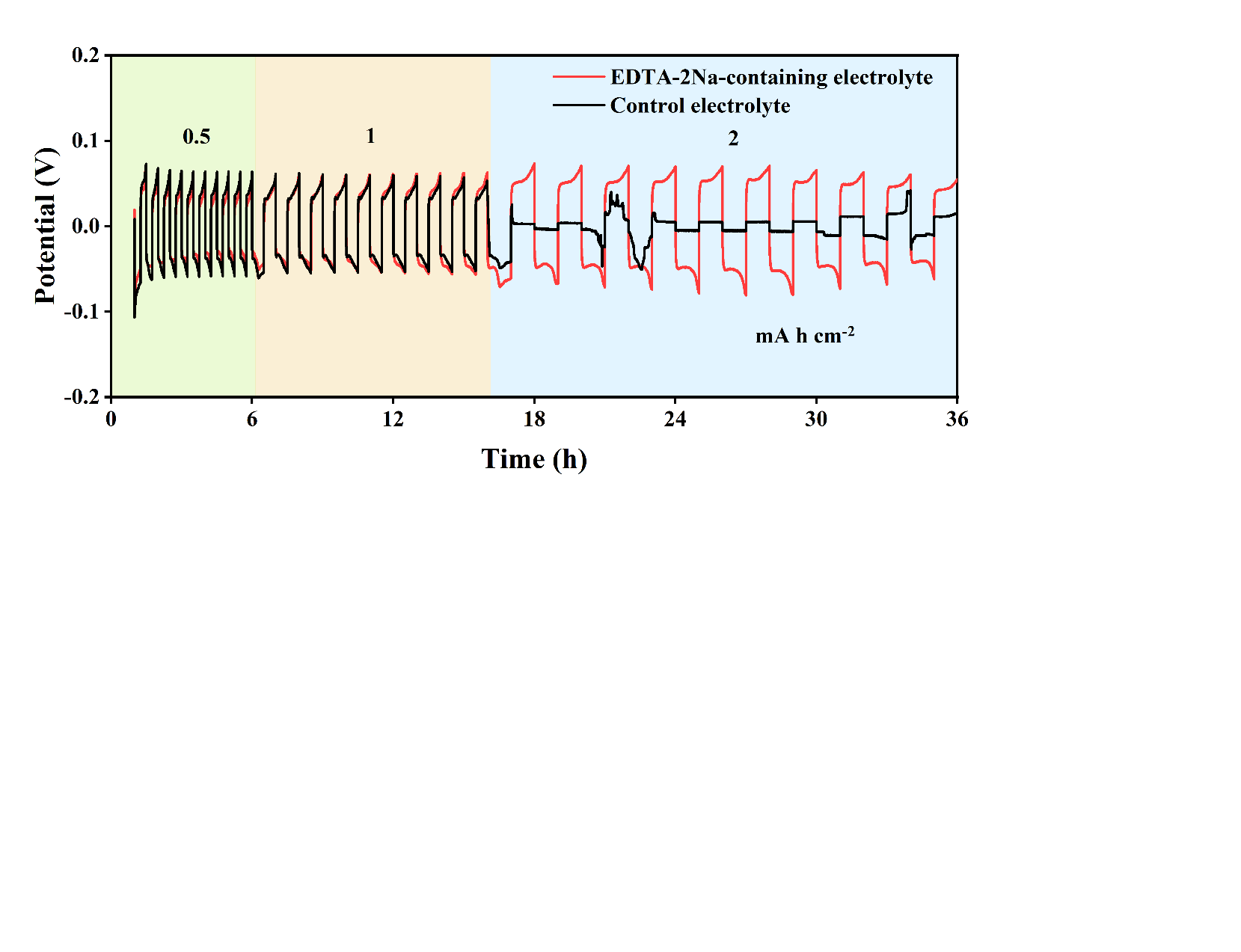
**

**Figure S5.** Galvanostatic Zn plating/stripping in Zn||Zn symmetrical cells at 2 mA cm^-2^ with different plating/stripping capacity.


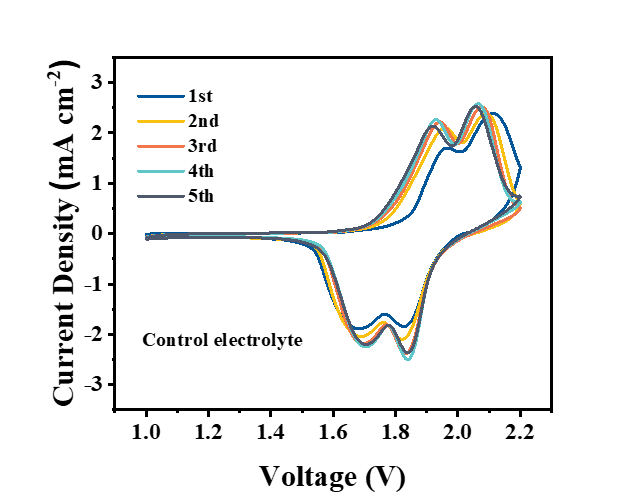


**Figure S6.** CV curves of Zn||LiMnO_4_ battery in the control electrolyte at 0.5 mV s^-1^.


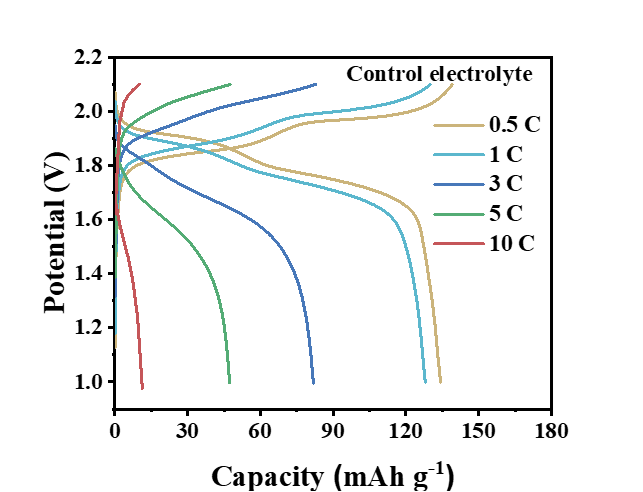


**Figure S7.** Typical charge/discharge curves for Zn||LiMnO_4_ cells in the control electrolyte with current density.

**Table S1.** The electrochemical performances of aqueous ZIBs using various additives.

| Electrolyte | Cathode | Cathode cyclability | Current density | Refs |
| --- | --- | --- | --- | --- |
| 1.26×10^-8^ M PbSO_4_ + 5% fumed silica + 1M Li_2_SO_4_ + 2M ZnSO_4_ | LiMn_2_O_4_ | 74.4% capacity retention after 300 cycles | 1 C | Mater. Today Energy 2017, 4, 34 |
| H_3_BO_3_ + 1M Li_2_SO_4_ + 1M ZnSO_4_ | LiMn_2_O_4_ | 78% capacity retention after 1000 cycles | 4 C | Chem. Eur. J. 2018, 24, 1667 |
| PAM/GO/EG | δ-MnO_2_ | 95.0% capacity retention after 100 cycles | 0.5 C | Front. Chem. 2020, 8, 603 |
| (NH_4_)_6_[Mo_7_O_24_]·4H_2_O + 1M ZnSO_4_ | NaV_3_O_8_·1.5H_2_O | 60% capacity retention after 500 cycles | 5 C | J. Mater. Chem. A 2021, 9, 7025 |
| Polyacrylamide + 0.1M MnSO_4_ | MnO_2_ | 87.2% capacity retention after 200 cycles | 0.8 C | Angew. Chem. 2019, 131, 15988-15994 |
| 5%EDTA-2Na + 1M ZnSO_4_ + 3M Li_2_SO_4_ | LiMn_2_O_4_ | 90.3% capacity retention after 150 cycles | 4 C | This work |
